# Peer-assisted teaching of basic surgical skills

**DOI:** 10.3402/meo.v20.27579

**Published:** 2015-06-03

**Authors:** Ryan Preece, Emily Clare Dickinson, Mohamed Sherif, Yousef Ibrahim, Ann Susan Ninan, Laxmi Aildasani, Sartaj Ahmed, Philip Smith

**Affiliations:** Cardiff University School of Medicine, Cardiff, Wales, UK

**Keywords:** clinical education, surgical education, peer-assisted learning, suturing, simulation

## Abstract

**Background:**

Basic surgical skills training is rarely emphasised in undergraduate medical curricula. However, the provision of skills tutorials requires significant commitment from time-constrained surgical faculty.

**Purpose:**

We aimed to determine how a peer-assisted suturing workshop could enhance surgical skills competency among medical students and enthuse them towards a career in surgery.

**Methods:**

Senior student tutors delivered two suturing workshops to second- and third- year medical students. Suturing performance was assessed before and after teaching in a 10-min suturing exercise (variables measured included number of sutures completed, suture tension, and inter-suture distance). Following the workshop, students completed a questionnaire assessing the effect of the workshop on their suturing technique and their intention to pursue a surgical career.

**Results:**

Thirty-five students attended. Eighty-one percent believed their medical school course provided insufficient basic surgical skills training. The mean number of sutures completed post-teaching increased significantly (*p*<0.001), and the standard deviation of mean inter-suture distance halved from ±4.7 mm pre-teaching, to ±2.6 mm post-teaching. All students found the teaching environment to be relaxed, and all felt the workshop helped to improve their suturing technique and confidence; 87% found the peer-taught workshop had increased their desire to undertake a career in surgery.

**Discussion:**

Peer-assisted learning suturing workshops can enhance medical students’ competence with surgical skills and inspire them towards a career in surgery. With very little staff faculty contribution, it is a cheap and sustainable way to ensure ongoing undergraduate surgical skills exposure.

There is increasing evidence from Europe and Australia that medical schools provide insufficient basic surgical skills (e.g., suturing) learning opportunities for undergraduates (1). Students often feel compelled to obtain exposure to these skills by attending (costly) extracurricular courses led by surgical societies and professional bodies.

Surgical trainees typically cite their undergraduate experiences of surgery as important in influencing career choice. Thus, a low level of medical student exposure to surgical practice could have an adverse impact on recruitment of future trainees (2). Enhancing undergraduate exposure to surgery through skills classes significantly increases student interest in surgery (3). However, these schemes, although effective, demand major time commitments from faculty members and surgical trainees.

Peer-assisted learning – an established teaching method whereby students in more senior years of a curriculum teach those in the earlier years (4) – is increasingly used in medical schools to provide students with basic surgical skills teaching. Already a proven effective method for teaching medical students topics such as anatomy (5), it has potential both to educate students and to increase their interest in pursuing a surgical career. Therefore, in this pilot study, we aimed to determine how a peer-assisted learning suturing workshop might enhance basic surgical skills amongst medical students and inspire them towards a career in surgery.

## Methods

We organised two 3-h peer-assisted learning suturing workshops for second- and third-year medical students at Cardiff University. The workshops were advertised to all second- and third-year students at the medical school. Students subsequently registered and attended the workshop on a voluntary basis. Prior to the session, student tutors attended the official ‘Surgical Skills for Students’ course run by the Royal College of Surgeons of England to acquire a sound knowledge of basic suturing technique.

The suturing workshop covered the principles of wound closure as well as basic knot tying and suturing techniques. Initially, we introduced students to each knot and suturing technique through a PowerPoint presentation. Subsequently, students repeatedly practised the skills on wound closure pads in small groups of three to four students with a designated student tutor.

### Suturing assessment

Both before and after teaching, we assessed students’ suturing ability. This involved them closing a 5-cm wound in a synthetic wound closure pad using an interrupted suturing technique. We set a time limit of 10 min for this task at which point we stopped the students and retrieved the pads. These were analysed by an investigator blinded as to whether the pad was sutured pre- or post-teaching using an online random code sequence generator programme (www.random.org).

For each suture pad, we measured the number of sutures completed, inter-suture distance, and suture tension using a previously described method (6) and made measurements using a millimetre Vernier caliper scale.

### Questionnaire

Following the workshop, students completed a questionnaire. This assessed their previous suturing experience, the impact of the workshop on their suturing technique, and their desire to pursue a surgical career. We scored their responses using a four-point Likert scale (1=strongly disagree, 2=disagree, 3=agree, 4=strongly agree) with space for free-text comments to the question ‘What was good about the workshop?’

### Statistical analysis

We used the *t*-test (independent samples and paired samples) to compare the mean suture tension and number of sutures completed before and after teaching. All statistical analyses were conducted using IBM SPSS Statistics version 20.

The Cardiff University School of Medicine Research Ethics Committee granted ethical approval for the study.

## Results

Thirty-five second- and third-year medical students attended the suturing workshop. All 35 (100%) students completed the suturing questionnaire and 29 (83%) participated in the suturing assessment.

### Suturing questionnaire

Before attending the suturing workshop, 43% of the students reported no experience or knowledge of suturing, 3% had read how to throw sutures but had not physically practised it, 51% had previously practised suturing in clinical skills laboratories, and 3% had actually performed suturing in a clinical setting on patients.

Twenty-six percent of students had previously attended an extra-curricular suturing course (provided by sources other than the medical school, e.g., surgical societies). Of these, two-thirds had attended 1–5 h of extracurricular suturing teaching and one-third had attended 6–10 h.

All students rated the quality of the skills course teaching as either good (11%) or very good (89%); none reported the teaching to be poor or very poor.

Table 1 shows student perceptions of the peer-assisted learning workshop and desires to pursue a surgical career. Table 2 shows free-text responses (from 31 students) to the question, ‘What was good about the workshop?’

**Table 1:** Student questionnaire responses

	Student response				
	
Questions	Strongly disagree (%)	Disagree (%)	Agree (%)	Strongly agree (%)	Mean±standard deviation
I have received enough basic surgical skills teaching at medical school	16	65	16	3	2.1±0.7
Before this teaching session I was considering undertaking a career in surgery	3	32	51	14	2.8±0.7
Learning surgical skills in this format makes me more interested in pursuing a career in surgery	0	11	63	26	3.1±0.6
This workshop has been useful for developing my technique and confidence of suturing	0	0	31	69	3.7±0.5
I would attend another peer-taught suturing workshop	0	0	34	66	3.7±0.5
I feel that I was taught in a relaxed environment	0	0	11	89	3.9±0.3

**Table 2:** Student perceptions of workshop strengths

Theme	Total number of related comments	Example
Qualities of tutors	17	‘Good enthusiastic teaching’
Teaching method	11	‘Very hands on’ ‘Very interactive’
Session length	8	‘Taught at good pace and able to practice a lot!’
Teaching environment	7	‘It was taught in a relaxing environment by students’
Group size	5	‘Really good teaching in small groups’
PowerPoint presentation	2	‘Good teaching in slides’
Equipment	1	‘Good equipment supplied’
Skills achieved	1	‘Able to learn how to perform two new stitches’

### Suturing assessment

Table 3 gives the results from the suturing assessment. The mean number of completed sutures significantly increased following teaching (*p*<0.001), although pre- and post-teaching mean suture tensions (*p* = 0.78) were similar. The standard deviation for the mean inter-suture distance nearly halved from ±4.7 mm pre-teaching to ±2.6 mm post-teaching.

**Table 3:** Suturing assessment measurements pre- and post-teaching

Suturing variable	Pre-teaching	Post-teaching	*p*
Mean number of sutures tied	2.3±1.6	5.3±1.7	0.001
Mean suture tension	16.9±17.0	16.1±20.9	0.78
Mean inter-suture distance (mm)	8.7±4.7	7.7±2.6	0.21

## Discussion

We have shown in this pilot study that senior medical students can effectively provide basic surgical skills learning opportunities to junior colleagues in an efficient manner independent of faculty contribution. Furthermore, we have identified that students enjoy the interactive and relaxed learning environment generated, such that involvement in peer-taught workshops reinforces their desire to pursue a surgical career.

This study is limited by its small sample size, containing a high proportion (65%) of self-selected students interested in pursuing a surgical career. Furthermore, including only second- and third-year students with variable surgical exposure might have pre-disposed to poor suturing technique pre-teaching. Nevertheless, our data show that a peer-taught suturing workshop can improve both undergraduate surgical skills and their interest in surgery.

Previous work has shown that UK medical schools provide only limited undergraduate skills training in knot-tying (17.4%) and suturing (24.7%) (1). A lack of undergraduate surgical skills training is the likely reason that senior students are often unprepared to perform suturing in clinical practice (7). Faculty-led basic surgical skills courses for undergraduates can increase skills dexterity and interest in surgery as a career (8) but inevitably draw surgical faculty from clinical practice (7). Peer-assisted learning has been investigated as a method to improve student dexterity and confidence with suturing (7); randomised trials show that senior medical students can provide surgical skills teaching as effectively as experienced surgical faculty (7).

Previous studies have not investigated student perceptions of peer-taught surgical skills, or the effect of these workshops on future intentions to pursue a surgical career. This is important, as cross-sectional data of general medical school cohorts suggest as few as 22% of students intend to pursue a surgical career (9). Furthermore, with a rising pressure for junior doctors to choose career pathways earlier, a lack of exposure to surgery may prompt fewer surgical training applications.

We identified that 81% of students felt they had received insufficient basic surgical skills teaching at medical school, a figure very similar to a previous study (1). All students felt that the suturing workshop helped to develop their technique and confidence with suturing. This was reflected by significant improvements in performance and consistency following the teaching. This supports earlier data in suggesting that peer-assisted learning is an effective format for enhancing undergraduate surgical skills competence (7).

Faculty-led surgical workshops for medical students have been shown to increase students’ desire to pursue a surgical career in 88% of students (3), but ours is the first to show this following a peer-assisted learning suturing workshop. Our data clearly suggest that students valued the peer-assisted learning format with all indicating that they would return for another student-led workshop. All students felt that they were taught in a relaxed environment and that they appreciated the enthusiastic, interactive, and relaxed session. The low-stress environment may have generated greater educational impact compared to faculty-led sessions, which sometimes are stressful for students, even to the point of being detrimental to learning (10).

The encouraging results of this pilot study justify a larger prospective investigation assessing both the retention of learnt skills over subsequent months (via further suturing assessment), and also the transferability to clinical practice of the surgical skills acquired.

## Conclusion

A peer-assisted learning suturing workshop is an effective educational tool, both to improve medical student competency with surgical skills and to inspire them to pursue a career in surgery. Furthermore, with the workshop being solely organised and delivered by medical students, this could prove a sustainable initiative to expose medical students to surgery without the need for significant input from time-constrained surgical faculty.
